# Supplementary material for: Feasibility and effectiveness of daily temperature screening to detect COVID-19 in a prospective cohort at a large public university
Source: BMC Public Health. 2021 Sep 16;21:1693. doi: 10.1186/s12889-021-11697-6 (PMC8445011; doi:10.1186/s12889-021-11697-6)
Supplement: Supplementary file 1 — Additional file 1: Supplemental Table 1. List of Symptoms Asked on Participant Daily Surveys. Supplemental Table 2. Answers to the Endline survey question “What was the most difficult or frustrating component of the study for you?”. Supplemental Figure 1. Number of surveys completed per day, all participants. Supplemental Figure 2. Number of surveys completed per day, students only (A) and faculty, staff, or essential workers only (B). [file 12889_2021_11697_MOESM1_ESM.docx]

**Supplemental Table 1. List of Symptoms Asked on Participant Daily Surveys.**

For purposes of this analysis, participants were considered "symptomatic" if on their daily survey they reported at least 3 symptoms from the list below, or any of the symptoms with "yes" in the second column.

| **Symptom** | **Sufficient to consider "symptomatic" if any symptom?†** |
| --- | --- |
| Dry cough (without mucus) | yes |
| Coughing up mucus | yes |
| Painful pressure in ears |  |
| Blocked nose |  |
| Runny nose |  |
| Sneezing |  |
| Watery eyes |  |
| Unusually hoarse voice |  |
| Fever | yes |
| Sweats | yes |
| Chills | yes |
| Headache |  |
| Tickles in the throat |  |
| Sore throat | yes |
| Muscle pain |  |
| Unusual pain or pressure in the chest |  |
| Painful sinuses (pain or pressure in the area above and below your eyes or behind your nose) |  |
| Swollen glands in your neck, armpits, or underneath your ears |  |
| Loss of appetite |  |
| Difficulty breathing | yes |
| Wheezing (a high-pitched whistling, or ratting sound when you breathe in or out) | yes |
| Shortness of breath | yes |
| Diarrhea |  |
| Stomach pain |  |
| Nausea |  |
| Vomiting |  |
| Unexplained trouble thinking or concentrating |  |
| Trouble sleeping |  |
| Fatigue (tiredness) |  |
| Loss of sense of taste | yes |
| Loss of sense of smell | yes |
| Eye pain |  |
| Swollen or red eyes |  |
| Body aches |  |
| Weakness |  |

† Symptoms with “yes” in the second column were considered to be symptoms commonly associated with SARS-CoV-2 infection.

**Supplemental Table 2. Answers to the Endline survey question “What was the most difficult or frustrating component of the study for you?”**

| **Component** | **n (%)** |
| --- | --- |
| Enrollment survey | 111 (5.2%) |
| Daily temperature monitoring | 766 (36.1%) |
| Daily symptom surveys | 157 (7.4%) |
| Weekly exposure surveys | 114 (5.4%) |
| Testing notifications triggered from survey responses | 33 (1.6%) |
| Random surveillance testing notifications | 71 (3.4%) |
| Communications with the study team (via text, email, phone) | 35 (1.7%) |
| Scheduling appointments at University Health Services (UHS) | 87 (4.1%) |
| Swab testing at UHS | 374 (17.6%) |
| Receiving swab results from UHS | 136 (6.4%) |
| Blood collection at UHS | 185 (8.7%) |
| Baseline participation payment (via Amazon gift card) | 50 (2.4%) |

**Supplemental Figure 1. Number of surveys completed per day, all participants**

**
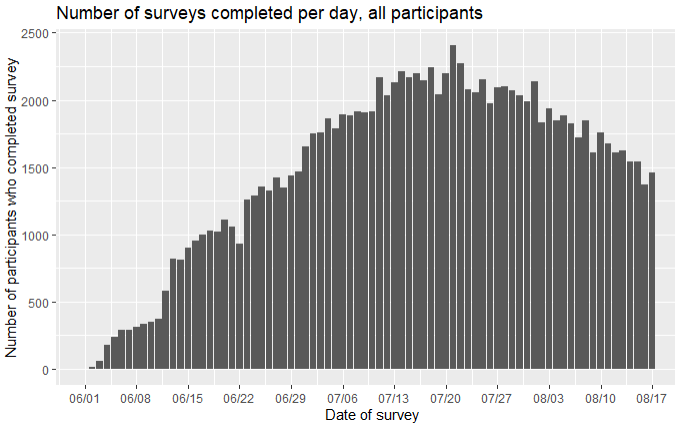
**

**Supplemental Figure 2. Number of surveys completed per day, students only (A) and faculty, staff, or essential workers only (B)**


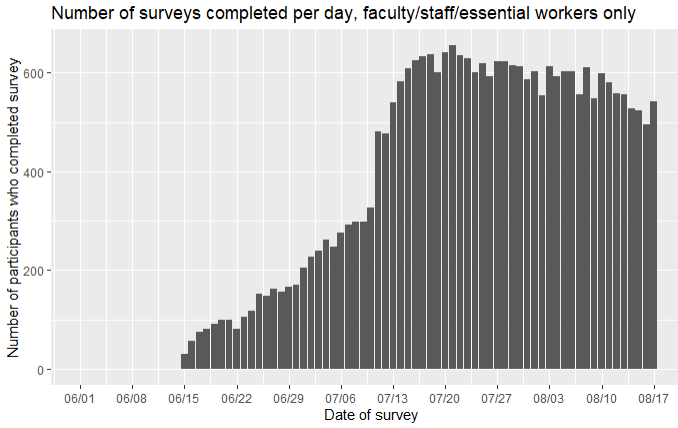

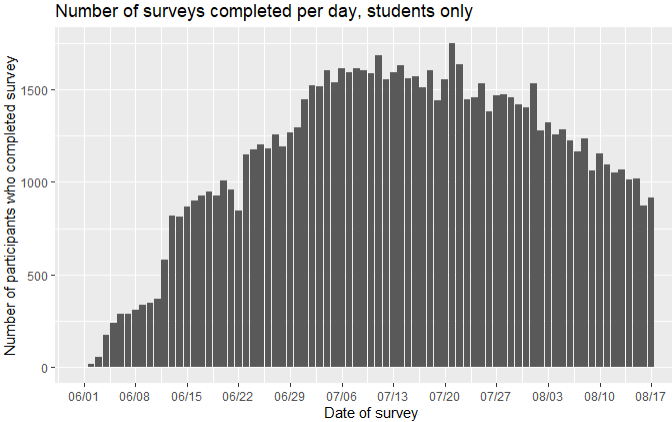


**A**

**B**
